# Patient-Tailored Augmented Reality Games for Assessing Upper Extremity Motor Impairments in Parkinson’s Disease and Stroke

**DOI:** 10.1007/s10916-018-1100-9

**Published:** 2018-10-30

**Authors:** Paulina J. M. Bank, Marina A. Cidota, P. (Elma) W. Ouwehand, Stephan G. Lukosch

**Affiliations:** 1 Department of Neurology, Leiden University Medical Center, PO Box 9600, 2300 RC Leiden, The Netherlands; 2 Faculty of Technology, Policy and Management, Delft University of Technology, Delft, The Netherlands; 3 Faculty of Mathematics and Computer Science, University of Bucharest, Bucharest, Romania

**Keywords:** Augmented reality, Engagement, Upper extremity, Motor function, Parkinson’s disease, Stroke

## Abstract

In clinical practice, upper extremity motor impairments are commonly assessed with disease-specific, subjectively scored and low-resolution rating scales that often do not consider the variations in tasks and environment that are essential aspects of daily life. Augmented reality (AR) systems with contactless tracking of the hand and upper body offer opportunities for objective quantification of motor (dys)function in a challenging, engaging and patient-tailored environment. In this study, we explore the potential of AR for evaluating 1) speed and goal-directedness of movements within the individually determined interaction space, 2) adaptation of hand opening to objects of different sizes, and 3) obstacle avoidance in healthy individuals (*N* = 10) and two highly prevalent neurological conditions (*N* = 10 patients with Parkinson’s Disease and *N* = 10 stroke patients). We successfully implemented three AR games to evaluate these key aspects of motor function. As expected, PD patients moved slower than controls and needed more time for task completion. No differences were observed between stroke patients and controls, perhaps because motor impairments in this patient group were relatively mild. Importantly, usability of our AR system was good and considerably improved compared to our previous study due to more natural and patient-tailored interaction. Although our findings testify to the potential of AR for assessing motor impairments in patients with neurological conditions and provide starting points for further improvement, there are still many steps to be taken towards application in clinical practice.

**Electronic supplementary material:**

The online version of this article (10.1007/s10916-018-1100-9) contains supplementary material, which is available to authorized users.

## Introduction

Objective assessment of upper extremity motor dysfunction is important for treatment selection and evaluation as well as monitoring disease progression in various neurological conditions (e.g. Parkinson’s Disease (PD), stroke). Motor impairments are commonly assessed with disease-specific, subjectively scored and low-resolution rating scales, or occasionally with cumbersome marker-based motion capture. Assessments are often limited to simple tasks, without considering variations in tasks and environment that are essential in daily life. Against this background, augmented reality (AR) systems with contactless tracking of the hand and upper body offer opportunities for objective quantification of motor (dys)function in a challenging, engaging and patient-tailored environment. AR gives clinicians full control over specific parameters (e.g. object size, movement distance) and allows for adjustment to individual capabilities. Importantly, it may result in more realistic behaviour than virtual reality [1], where immersion in a completely synthetic world may interfere with natural behaviour.

AR systems have successfully been developed for rehabilitation of upper extremity motor function, using ‘exergames’ in virtual worlds to motivate [2] and engage patients to perform repetitive tasks [3]. Thereby, a variety of interaction methods have been used (such as gloves [4, 5], real objects [6–10], markers attached to the hand or arm [1, 11] or contactless tracking [12–14]) in combination with different visualisation styles (such as monitors [5, 7, 12, 13] and 2D or 3D rendering in the direct environment of the patient (2D: [8, 9, 10, 11, 14], 3D: [4, 6])). None of these AR systems uses contactless tracking in combination with 3D rendering in the direct environment of the patient. Moreover, research on AR for quantitative assessment of upper extremity motor function is scarce [15]. Especially for assessment, it is important that patients are able to naturally interact with virtual content (e.g., without being restricted by gloves, markers or wires attached to the hand) in the 3D personal space where most daily life activities take place. In the present study, we therefore combined contactless tracking with 3D rendering in the direct environment and designed three games [16] to evaluate 1) speed and goal-directedness of movements within the individually determined interaction space, 2) adaptation of hand opening to objects of different sizes, and 3) obstacle avoidance. In doing so, we aim to exploit the potential of AR (in terms of engagement and flexibility) to assess key aspects of upper extremity motor function in healthy individuals and two highly prevalent neurological conditions (PD, stroke). Based on our previous study [15], we aimed to improve usability by providing more natural and patient-tailored interaction as well as enlarging interaction space.

## Materials and methods

### Participants

Ten PD patients, ten stroke patients (>12 weeks post-stroke) and ten age-matched healthy controls participated in this study (Table 1). Patients were recruited from outpatient clinics of the Department of Neurology and Department of Rehabilitation Medicine of the Leiden University Medical Center. Patients were able to lift their arms above shoulder level and had no additional disorders of the central nervous system or other conditions that could affect upper extremity function. Controls had normal or corrected to normal vision, had no apparent cognitive disorders or deficits, and had no history of disorders affecting upper extremity function. Written informed consent was obtained from all participants. The ethical committee of the Leiden University Medical Center approved the protocol.

**Table 1:** Participant characteristics

*N*	Controls	PD patients	Stroke patients
	10	10	10
Sex (male/female)	6 / 4	6 / 4	6 / 4
Age (yr) (mean, SD) ^a^	61.6 ± 6.8	60.8 ± 7.5	60.5 ± 7.0
Disease duration (yr) (median, IQR)	–	11.9 [7.4–15.7]	3.5 [1.9–9.1]
Tested side (dominant/non-dominant)	5 / 5	6 / 4	6 / 4
MoCA (median, IQR) ^b^	28.5 [27.5–29.3]	27.5 [25.8–29.3]	26.0 [24.8–27.3] *
PD-specific clinical characteristics			
Stereotactic surgery (yes/no)	–	3 / 7	–
Hoehn and Yahr stage (median, range) ^c^	–	2 [1–3]	–
SPES/SCOPA total score (mean, SD) ^d^	–	18.1 ± 4.6	–
Stroke-specific clinical characteristics			
First ever stroke (%)	–	–	90
Type of stroke (ischemic/hemorrhage)	–	–	9 / 1
Lesion side (left/right/both)	–	–	6 / 4 / 0
Modified Rankin Scale (median, range) ^e^	–	–	1.5 [1–3]
Fugl-Meyer Upper Extremity Scale (median, IQR) ^f^	–	–	59.5 [55.8–64]

^a^ Not significantly different between PD patients and controls (*t*(18) = 0.25, *P* = .81) or between stroke patients and controls (*t*(18) = 0.37, *P* = .71). ^b^ MoCA = Montreal Cognitive Assessment; 0–30; high: better [24]; * significantly reduced compared to controls (*P* = .01). One control, two PD patients, and four stroke patients scored <26 (cutoff for mild cognitive impairment); ^c^ 0–5; high: worse [17]; ^d^ SPES/SCOPA motor examination, total score: 0–63; high: worse [18]; ^e^ 0–5; high: worse [19]; ^f^ 0–66; high: better [20]; range of observed scores = 43–65

### Measurement instruments and data collection procedure

#### Questionnaires

After each game, task load was evaluated using the NASA-TLX questionnaire (1–7; high: worse [21]) and engagement was evaluated using a subset of 14 questions (1–5; high: better; [15]) from the Game Experience Questionnaire (GEQ; [22, 23]). At the end of the experiment, the System Usability Scale (SUS; 0–100; high: better; [24]) and a questionnaire on presence (1–7; high: better; Online Resource 1, adapted from [25]) were used to evaluate user experiences and identify opportunities for further improvement.

#### Hardware

**Fig. 1:**
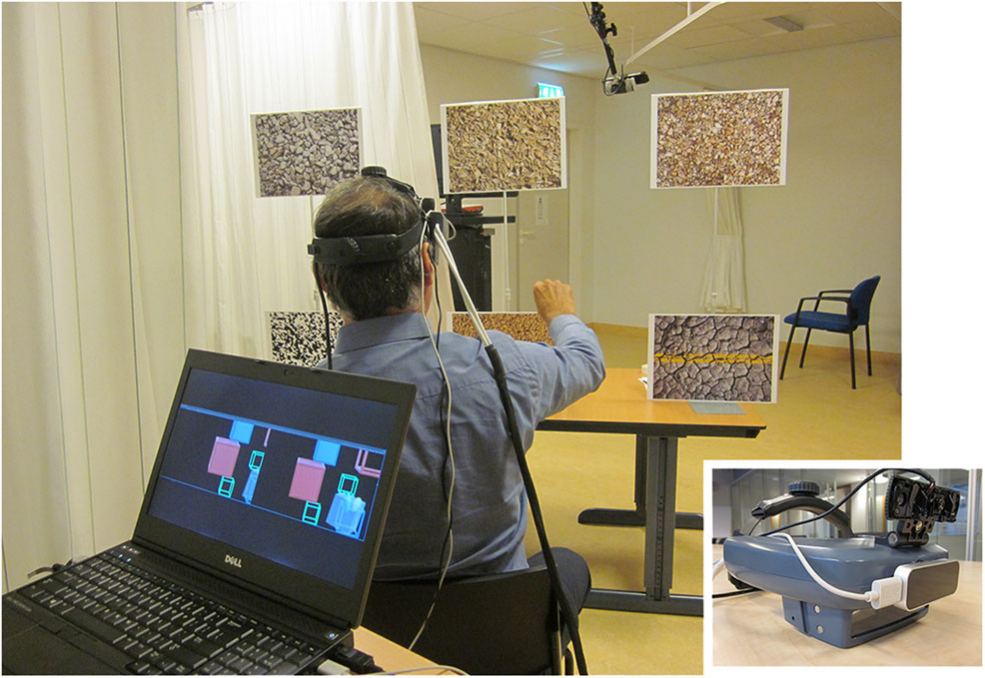
Impression of a participant during the experiment, with a close-up view of the optical see-through HMD with Leap Motion and webcam mounted on top of it. The laptop screen displayed a copy of the stereo images that were presented to the participant’s left and right eye in the HMD

Virtual content was visualized using an AIRO II head-mounted display (HMD) (Fig. 1; Cinoptics, Maastricht, The Netherlands) with Leap Motion for contactless tracking of the hand (Leap Motion Inc., San Francisco, CA, USA) and Logitech C922 Pro Stream Webcam for marker recognition. A Microsoft Kinect™ v2 sensor was placed at 3 m from the participant, at an angle of 45° to the left side to avoid occlusion by markers. The application was run on a Dell Precision M4800 laptop.

#### Software

Software components were integrated in Unity3D (version 5.6.0, Unity Technologies, San Francisco, USA). The virtual world was aligned to the real world using the Vuforia tracking library (version 6.2.6, PTC Inc., Needham, USA). The Leap Motion Orion Beta software development kit (SDK) provided 3D-coordinates of hand ‘joints’ (e.g., hand palm and finger tips). The Kinect for Windows SDK (version 2.0) provided 3D-coordinates of body points (e.g., wrist, elbow and shoulder). The following sections provide a brief description of each game. Technical details and illustrative movies are provided in Online Resources 2–5.

#### Game 1: Balloons

**Fig. 2:**
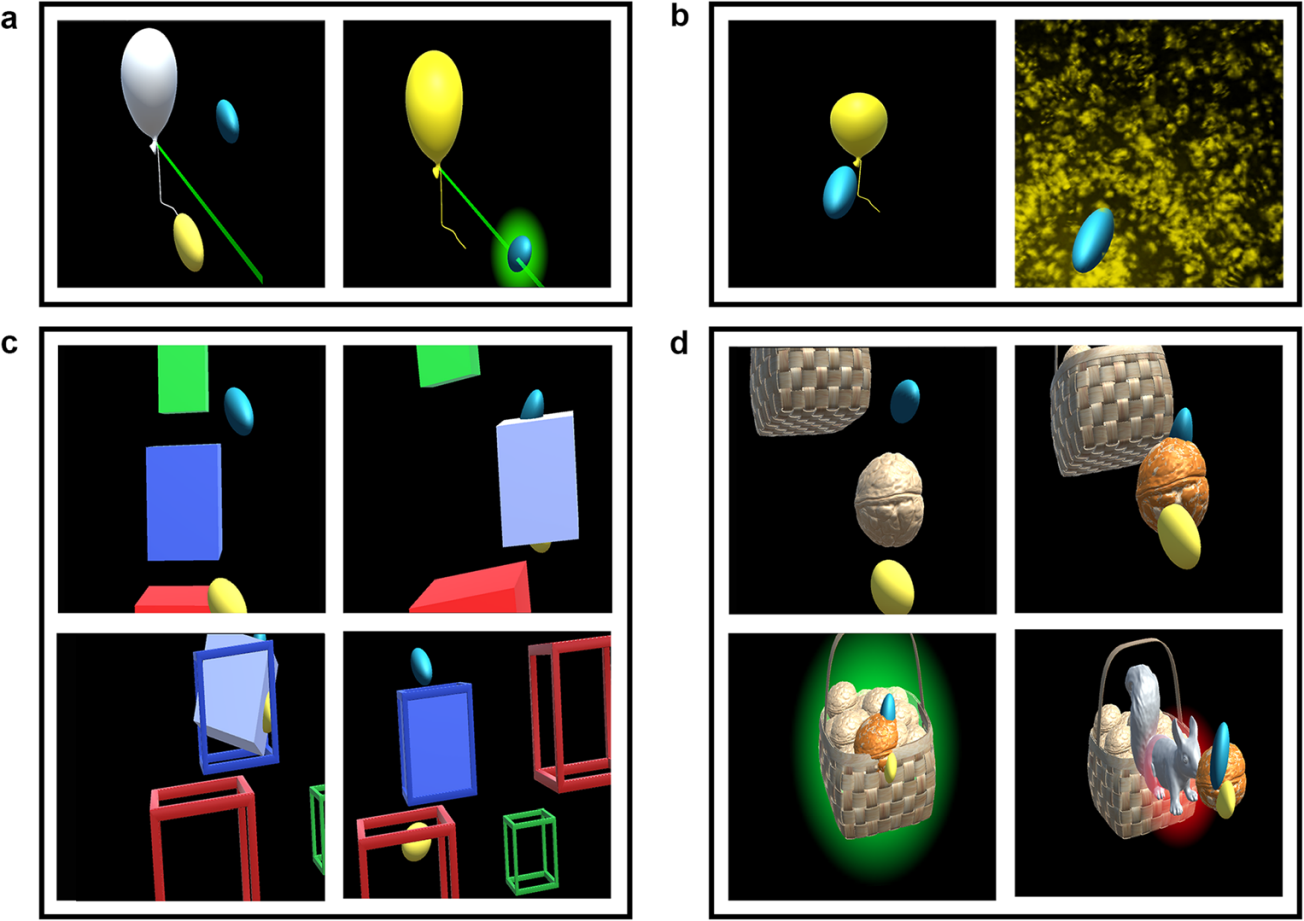
Impression of the three AR games. (**a**) first part of game 1: “Balloons”; (**b**) second part of game 1: “Balloons”; (**c**) game 2: “Melody cubes”; (**d**) game 3: “Hungry squirrel”. The dark grey background of these screen captures appears almost transparent in the HMD so that the participant’s real hand and real environment are visible together with the virtual content

The ‘Balloons’ game (illustrated in Figs. 2a and b) aimed to evaluate the speed and goal-directedness of movements within the individually determined interaction space. First, interaction space was determined from the furthest points of intersection between the index finger and a virtual line from the participant’s estimated shoulder position towards a faraway balloon (positioned at the ipsilateral/contralateral side of the body and above/below the shoulder). Participants were instructed to reach as far as possible in the indicated direction while keeping the trunk against the backrest of the seat. Next, twelve balloons were displayed at various positions within the measured interaction space and at random depths (one at a time, in predefined order). Participants were instructed to touch the balloons as quickly as possible. Balloons exploded upon touch or disappeared if not touched within 20 s.

#### Game 2: Melody cubes

The ‘Melody Cubes’ game (illustrated in Fig. 2c) aimed to evaluate whether virtual objects of different sizes induce adaptation of hand opening and affect the quality of object manipulation. Twelve opaque cubes (four of each size: 5, 7.5, and 10 cm) had to be moved from a stock pile located contralateral to the tested arm to twelve empty cubes placed at various positions within the individually determined interaction space. When the cube touched a size- and colour-matched empty cube, cube placement was considered successful and a 12th part of a well-known melody was played. Once all empty cubes were filled, the complete melody was played as reward for completing the game.

#### Game 3: Hungry squirrel

The ‘Hungry Squirrel’ game (illustrated in Fig. 2d) aimed to evaluate obstacle avoidance during goal-directed movements. Sixteen walnuts had to be put into a virtual basket that was alternately positioned in the upper or lower half of the interaction space (at maximum depth; 4 practice trials followed by 12 test trials). At the beginning of each trial, one walnut appeared between the participant’s shoulder and the basket (40 cm from the basket, or at least approximately 35 cm from the shoulder). During practice and in 4 out of 12 test trials, there was no obstacle (NO). In 8 out of 12 test trials, an obstacle (virtual squirrel) was positioned halfway between the walnut’s initial position and the basket. This squirrel was either visible from the start of the trial (visible obstacle, VO; 4x) or appeared after the participant started moving the walnut (surprise obstacle, SO; 4x), i.e., as soon as the walnut was within a specific distance from the squirrel’s position (patient-tailored: based on movement speed during the final two practice trials). Each scenario was presented once within a block of three trials to ensure an even distribution within the game. Visual feedback was provided when the walnut was placed into the basket (green glow) or when it touched the squirrel (red glow).

#### General data collection procedure

Participants sat in a chair without armrests. After assessment of reachable workspace (methods and results in Online Resource 6), participants familiarized themselves with the HMD and interaction with virtual cubes. Each AR game was explained using printed screen captures. Game 1 was performed first, because positions of virtual objects in the other games were based on the individually determined interaction space. The order of games 2 and 3 was counterbalanced between participants. Patients performed all tasks with their (most) affected arm. Controls performed all tasks with their dominant arm (*N* = 5) or non-dominant arm (*N* = 5). In all games, visual feedback was provided to facilitate interaction with the virtual content: ellipsoids represented the tips of the virtual index finger and thumb [15, 26], virtual objects changed colour during interaction, and a visual cue was presented when the object of interest was located outside view of the HMD. Total duration of the experiment (including clinical assessments and questionnaires) ranged from 35 to 105 min.

##### Data accessibility statement

Supporting data are made available from the 4TU.ResearchData repository (10.4121/uuid:81b7bfcb-47db-42e7-bf38-9560a376b8d5).

### Data analysis

Data was processed using MATLAB (The Mathworks Inc., Natick MA, USA, version R2016a).

#### Game 1: Balloons

*Maximum reach distance (MRD)* per quadrant was calculated as maximum distance between index finger and shoulder in each movement direction, expressed as percentage of Kinect-extracted upper extremity length (i.e., length of upper arm + forearm + hand including index finger) to allow comparison between individuals. From the second part of the game, *success rate* was calculated as percentage of balloons touched within 20 s. Speed of reaching movements was evaluated by means of *T*_*balloon*_ (i.e., time in seconds from balloon appearance until touch) and average *movement speed* (in cm/s; based on ‘index finger tip’ coordinate provided by Leap Motion), while goal-directedness was evaluated by means of *relative path length* (dimensionless; index finger’s actual path length divided by shortest possible distance, from the moment that the balloon became visible in the HMD until it was touched).

#### Game 2: Melody cubes

Hand opening was quantified as linear distance between tips of the thumb and index finger, for initial grasp (*HO*_*initial*_) and on average during interaction (*HO*_*interaction*_). Quality of object manipulation was evaluated by means of *T*_*cube*_ (i.e., time in seconds from first interaction until correct placement), *number of interaction episodes*, and average *movement speed* during interaction (in cm/s; based on ‘hand palm’ coordinate provided by Leap Motion).

#### Game 3: Hungry squirrel

*Success rate* was calculated for each scenario as the percentage of trials in which the walnut was put into the basket without touching the squirrel. Further analyses were limited to scenarios NO and VO, because the obstacle appeared in only 62% of trials in scenario SO due to unforeseeable changes in movement trajectory. Performance was evaluated by means of *T*_*walnut*_ (time in seconds from first interaction until touching basket or squirrel), average *movement speed* during interaction (in cm/s; based on ‘hand palm’ coordinate provided by Leap Motion) and, in case of successful obstacle avoidance, *relative path length* (dimensionless; walnut’s actual path length divided by shortest possible distance from position at first grasp to final position in basket).

### Statistical analysis

For each participant, aggregated scores were computed for each questionnaire (for NASA-TLX, presence, and engagement: mean value of all items (using inverted score for selected items), for SUS: as described in [24]). Median values were used for *T*_*balloon*_, *T*_*cube*_ and *T*_*walnut*_ and for the number of interaction episodes to reduce the influence of outliers. All other dependent variables were averaged over balloons (game 1), per cube size (game 2) or per target position per scenario (game 3) for each participant. Statistical analyses were performed using IBM® SPSS® Statistics 23.0 (IBM Corp., Armonk NY). For success rate (games 1 and 3) deviations from normality could not be resolved by transformations. ^10^log transformation was applied to inverted values of MRD (i.e.,100-MRD), to *T*_*balloon*_, *T*_*cube*_ and *T*_*walnut*_ and to relative path length (game 1 and 3) prior to statistical analysis (for reasons of clarity, untransformed data are presented in Results).

We did not intend to directly compare outcome parameters between the two patient groups. For all outcome measures, separate statistical analyses were therefore conducted to compare either PD patients versus controls and stroke patients versus controls. MRD was submitted to mixed analyses of variance (ANOVAs) with group as between-subjects factor (PD vs. control or stroke vs. control) and quadrant as within-subjects factor (ipsilateral upper, ipsilateral lower, contralateral upper, contralateral lower). For game 1, success rate was compared between groups using Mann-Whitney *U*-tests (PD vs. control, stroke vs. control). Other outcome measures were compared between groups using *t*-tests (PD vs. control, stroke vs. control). For game 2, outcome measures were submitted to mixed ANOVAs with group (PD vs. control or stroke vs. control) as between-subject factor and cube size (5 cm, 7.5 cm, 10 cm) as within-subjects factor. For game 3, success rate of scenario VO was compared between groups (using Mann-Whitney *U*-tests; PD vs. control, stroke vs. control) and to scenario NO (using a one-sample Wilcoxon signed-rank test, test value: 100%). Other outcome measures were submitted to mixed ANOVAs with group (PD vs. control or stroke vs. control) as between-subject factor and scenario (NO vs. VO) and target position (upper vs. lower) as within-subjects factors. Usability (SUS) and presence were compared between groups using *t*-tests (PD vs. control, stroke vs. control). Task load (NASA-TLX) and engagement (GEQ-subset) were submitted to mixed ANOVAs with group (PD vs. control or stroke vs. control) as between-subject factor and game as within-subjects factor. We also explored associations between user experiences and selected AR outcomes (Online Resource 7). For all ANOVAs, degrees of freedom were adjusted if the sphericity assumption was violated [27]. Significance was set at *P* < .05, with Bonferroni correction for follow-up analyses. All values are presented as mean ± standard deviation, except for success rate (values presented as median [interquartile range]).

## Results

**Fig. 3:**
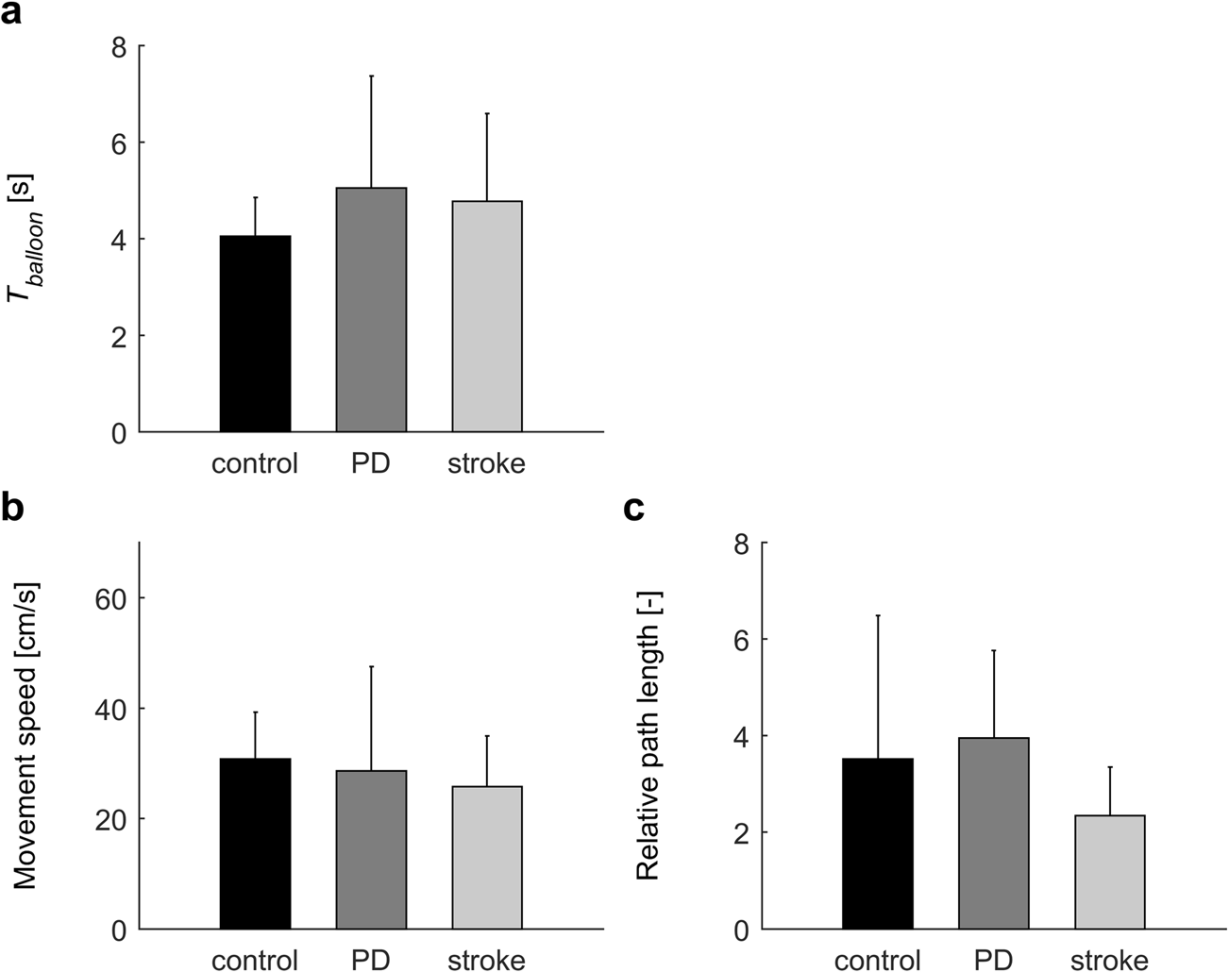
Results of the second part of game 1: “Balloons”. Error bars represent standard deviations. No significant group differences were detected

**Fig. 4:**
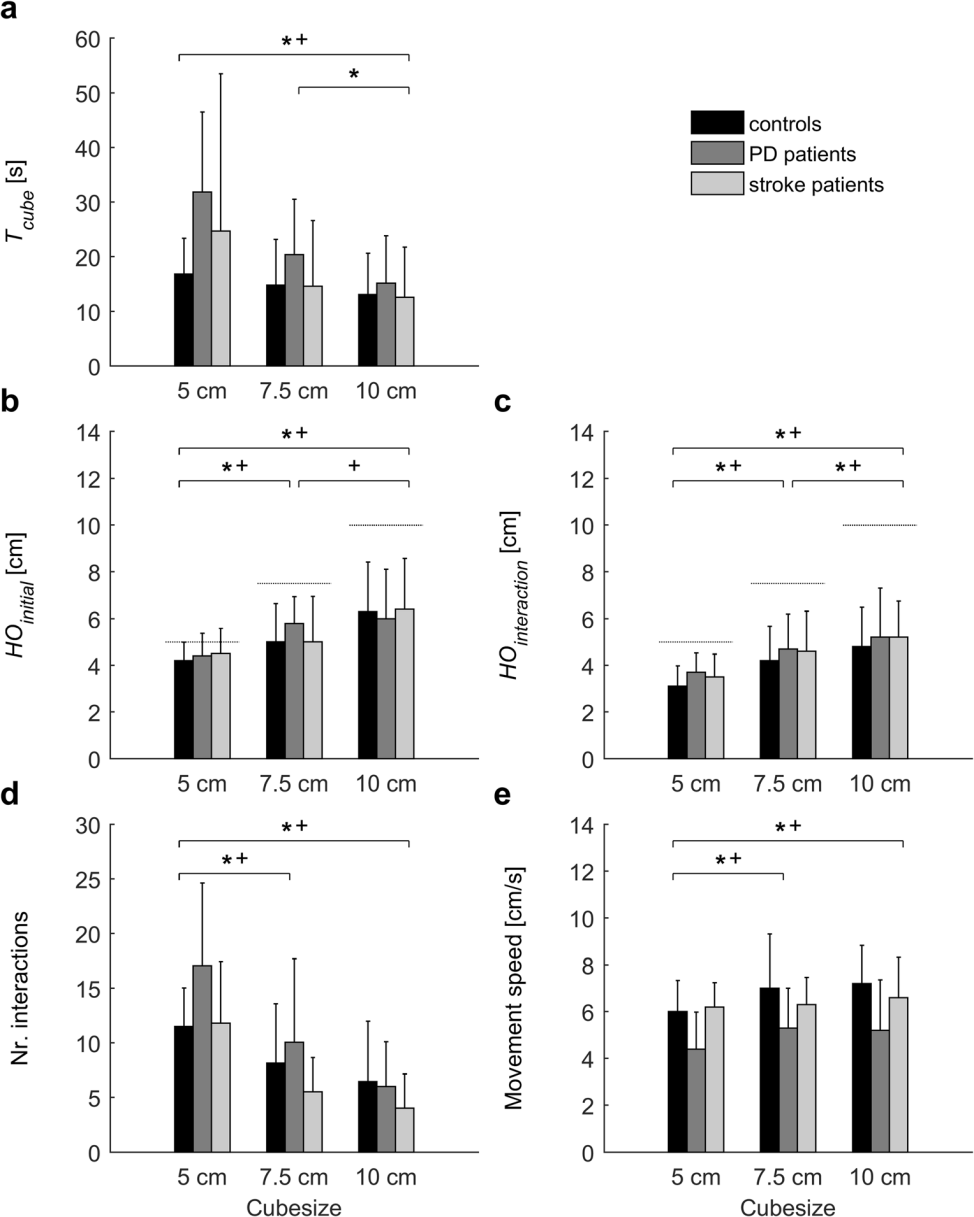
Results of game 2: “Melody cubes”. Error bars represent standard deviations. Symbols indicate significant differences (*P* < .05) between cube sizes (* PD/control analysis, ^+^ stroke/control analysis). Dotted lines in panels **b** and **c** indicate the actual size of the virtual cubes. Only for movement speed (panel **e**) a significant effect of group was observed, with PD patients moving slower than controls

**Fig. 5:**
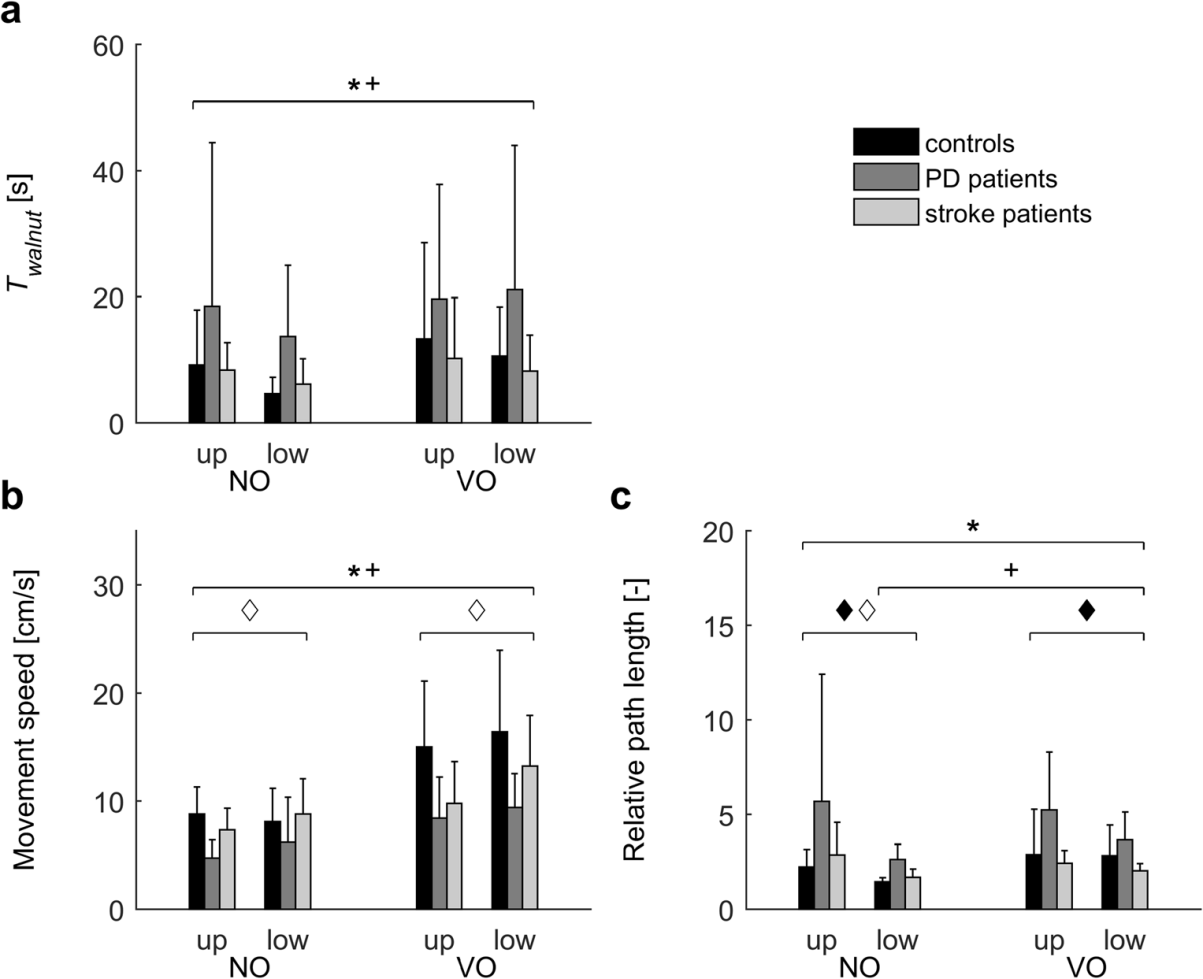
Results of game 3 “Hungry squirrel”. Error bars represent standard deviations. Symbols indicate significant differences (*P* < .05) between scenarios (NO: no obstacle vs. VO: visible obstacle; * PD/control analysis, ^+^ stroke/control analysis), and between target positions (upper vs. lower; ♦ PD/control analysis ◊ stroke/control analysis). For all three outcome measures a significant effect of group was observed: compared to controls, PD patients needed more time (panel **a**), moved slower (panel **b**) and had a longer relative path length (panel **c**). Only in stroke patients the relative path length was not affected by the presence of an obstacle (panel **c**)

Table 2 presents significant results for game outcome measures and user experiences. Results of associated post-hoc analyses are presented in Figs. 3, 4 and 5 and described in the following sections.

**Table 2:** Significant statistical results for game outcome measures and user experiences

Outcome	Effect	PD patients versus controls			Stroke patients versus controls		
		Test statistic	*P*	Effect size	Test statistic	*P*	Effect size
Game 1: Balloons							
Maximum reach distance (MRD) [%]^a,d^	Q	*F*(3,51) = 4.6	.007	.21	*F*(3,51) = 4.0	.01	.19
	G	*F*(1,17) = 5.1	.04	.23	–		
Success rate [%] ^e^	G	–			–		
T_balloon_ [s]^f,g^	G	–			–		
Movement speed [cm/s]^f^	G	–			–		
Relative path length [–]^f, g^	G	–			–		
Game 2: Melody Cubes							
*T*_*cube*_ [s] ^d,g^	CS	*F*(2,36) = 14.3	<.001	.44	*F*(2,36) = 4.4	.02	.20
	G	–			–		
*HO*_*initial*_ [cm] ^d^	CS	*F*(2,36) = 22.0	<.001	.55	*F*(2,36) = 20.8	<.001	.54
	G	–			–		
*HO*_*interaction*_ [cm] ^d^	CS	*F*(2,36) = 22.8	<.001	.56	*F*(2,36) = 28.0	<.001	.61
	G	–			–		
Movement speed [cm/s] ^d^	CS	*F*(2,36) = 5.3	.01	.23	*F*(2,36) = 4.3	.02	.19
	G	*F*(1,18) = 6.5	.02	.27	–		
Number of interaction episodes ^d^	CS	*F*(2,36) = 13.6	<.001	.43	*F*(2,36) = 14.9	<.001	.45
	G	–			–		
Game 3: Hungry Squirrel							
Success rate [%] ^e^	G	–			–		
*T*_*walnut*_ [s] ^b,d,g^	S	*F*(1,18) = 7.8	.01	.30	*F*(1,17) = 7.2	.02	.30
	G	*F*(1,18) = 4.8	.04	.21	–		
Movement speed [cm/s] ^b,d^	S	*F*(1,18) = 29.0	<.001	.62	*F*(1,17) = 22.9	<.001	.57
	TP	–			*F*(1,17) = 4.7	.04	.22
	G	*F*(1,18) = 10.7	.004	.37	–		
Relative path length [−] ^b,d,g^	S	*F*(1,15) = 11.8	.004	.44	*F*(1,16) = 8.6	.01	.35
	TP	*F*(1,15) = 6.3	.02	.29	*F*(1,16) = 6.9	.02	.30
	S × TP	–			*F*(1,16) = 7.4	.02	.32
	G	*F*(1,15) = 11.5	.004	.43	–		
	S × G	–			*F*(1,16) = 4.9	.05	.22
User experiences							
Workload (NASA-TLX) ^d^	GA	*F*(2,36) = 4.1	.03	.19	–		
	G	–			–		
Engagement (GEQ-subset) ^d^	GA	*F*(2,36) = 5.0	.01	.22	–		
	G	–			–		
Usability (SUS) ^f^	G	–			–		
Presence ^f^	G	–			–		

G = group (PD vs. control, stroke vs. control; as indicated); Q = quadrant (ipsilateral upper, ipsilateral lower, contralateral upper and contralateral lower; only for maximum reach distance); CS = cube size (5 cm, 7.5 cm, 10 cm; only for game 2); S = scenario (no obstacle (NO) vs. visible obstacle (VO); only for game 3); TP = target position (upper vs. lower; only for game 3); GA = game (1, 2, 3; only for questionnaires on user experience). Comparisons were based on *N* = 10 controls, *N* = 10 PD patients and *N* = 10 stroke patients, unless indicated otherwise. ^a^ Based on *N* = 10 controls, *N* = 9 PD patients and *N* = 9 stroke patients, due to technical issues with Kinect; ^b^ Based on *N* = 10 controls, *N* = 10 PD patients and *N* = 9 stroke patients, because no data was available for the upper target (scenario VO) for one stroke patient who skipped the final five movements towards the upper target (too burdensome); ^c^ Based on *N* = 9 controls, *N* = 8 PD patients and *N* = 9 stoke patients, who successfully avoided at least one obstacle per target position in scenario VO; ^d^ Mixed ANOVAs, effect size quantified as partial eta squared (*η*_*p*_^*2*^); ^e^ Mann-Whitney *U*-tests; ^f^ Independent *t-*tests; ^g^ values were ^10^log transformed for statistical analysis

### Game 1: Balloons

MRD tended to be slightly greater in controls (98.0 ± 2.9%) than in PD patients (96.8 ± 2.9%, *P* = .04) and stroke patients (95.5 ± 2.9%, *P* = .06). MRD tended to be greater for the ipsilateral upper quadrant (mean ± SD for all participants: 98.0 ± 1.9%) than for the ipsilateral lower quadrant (96.6 ± 3.2%, *P* < .06) and the contralateral lower quadrant (97.0 ± 2.5%, *P* < .07), but not compared to the contralateral upper quadrant (95.4 ± 10.3%, *P* > .19). Success rate was not different between controls (100 [100–100]%) and PD patients (100 [92–100]%, *P* = .58) or stroke patients (100 [90–100]%, *P* = .58). Also *T*_*balloon*_, movement speed, and relative path length were not significantly different between groups (Fig. 3).

### Game 2: Melody cubes

*HO*_*initial*_ and *HO*_*interaction*_ increased with cube size, but actual hand opening did not exactly match the size of the virtual cubes (Figs. 4b and c). *HO*_*interaction*_ was smaller than *HO*_*initial*_ for all three cube sizes (*t*(29) > 5.79, *P* < .001). Quality of object manipulation also depended on cube size: the smaller the cube, the higher *T*_*cube*_ (Fig. 4a), the more interaction episodes (Fig. 4d; indicating more frequent loss of interaction) and the lower movement speed (Fig. 4e). The only significant difference between groups was observed for movement speed: PD patients moved slower than controls.

### Game 3: Hungry squirrel

Success rate in scenario VO did not differ between controls (100 [100–100]%) and PD patients (100 [75–100]%, *P* = .21) or stroke patients (100 [75–100]%, *P* = .09). Overall, success rate was lower in scenario VO (100 [94–100]%) than in scenario NO (100% for all participants; *P* = .01). The presence of an obstacle affected all outcome measures (Table 2, Fig. 5): participants needed more time (all groups), movements were slower (all groups), and relative path length was longer (PD patients, controls). Compared to controls, PD patients needed more time, their movements were slower, and relative path length was longer. No significant differences were observed between stroke patients and controls.

### User experiences

Task load and engagement did not differ between games (post-hoc analyses: *P* > .05). No group differences were observed for task load (overall mean score: 3.2 ± 1.0 out of 7), engagement (3.8 ± 0.5 out of 5), presence (4.4 ± 0.7 out of 7), or usability (69.3 ± 13.7 out of 100). 53% of all participants scored above the threshold value (68) for good usability [24].

## Discussion

We successfully implemented three AR games using an optical see-through HMD and contactless hand and body tracking to evaluate key aspects of motor function. While our results testify to the potential of AR, there are still many steps to be taken towards application in clinical practice.

Speed and goal-directedness of movements (game 1), adjustment of hand opening to virtual objects of different sizes (game 2) and obstacle avoidance (game 3) were comparable between patients and controls. As expected, PD patients moved slightly slower than controls [28], but only significantly for games 2 and 3. No differences were observed between stroke patients and controls, perhaps because motor impairments in this patient group were relatively mild.

Individual assessment of interaction space (first part of game 1) allowed for patient-tailored positioning of virtual objects in all other games (i.e., dependent on arm length and motor abilities). Still, the so obtained MRD cannot be considered a proxy for the 3D upper extremity reachable workspace (Online Resource 6). There appeared to be ceiling effect for MRD in our sample of healthy controls and relatively mildly affected PD patients and stroke patients, with MRD being close to 100% in all directions and only very small (albeit significant) differences between groups. The limited field of view of the HMD and space restrictions for aligning virtual content to the real world (despite the use of multiple markers) hampered evaluation of MRD in the ‘extreme’ directions where limitations may be more evident. The second part of game 1 appeared not sensitive enough to detect a reduction of speed and goal-directedness of movements in PD patients (as was expected and observed in game 2 and 3), suggesting that its difficulty needs to be increased (e.g. by reducing balloon size to increase precision demands).

Although hand opening in game 2 depended on cube size, it did not exactly match the size of the virtual cubes. Most participants placed their thumb and index finger on opposite sides of the cube, but criteria for interaction (see Online Resource 2) also allowed participants to grasp the cube at a corner − a strategy that would be impossible when interacting with a real object subjected to gravity. The smallest cube appeared most difficult to manipulate, consistent with higher precision requirements for grasping small objects [29] but perhaps partly due to the fact that interaction margins were proportional to cube size (and were thus smallest in absolute terms). Future applications have to implicitly ‘force’ participants to better match hand opening to cube size and apply a realistic grasp. It has to be noted, however, that too strict criteria for interaction will result in frequent loss of interaction, which in turn negatively affects usability and user experience [15]. Although virtual object manipulation may benefit from haptic feedback [30], available solutions (e.g. instrumented gloves, exoskeletons) are expensive and may restrict freedom of movement (e.g. [31, 32]). In our study, contactless hand tracking in combination with visual feedback of thumb and index finger appeared sufficient for virtual object manipulation.

As expected, the presence of an obstacle in game 3 led to increased relative path length, slower movement, and longer task completion time. Obstacles visible from the beginning (VO) were successfully avoided in almost 100% of trials. Unfortunately, implementation of the ‘surprise obstacle’ (SO) was not successful due to between-trial variations of movement speed in combination with the relatively short movement distance (limited by a minimum distance from HMD and maximum reaching distance), which made it almost impossible to present the obstacle *after* movement initiation *and* allow enough time for avoidance. Some participants moved around the invisible ‘trigger zone’ for obstacle appearance (obstacle appeared in only 38% of SO trials). This happened also in some NO trials, thereby reducing differences between NO and obstacle scenarios.

In addition to game-specific considerations that have been addressed in previous sections, some general limitations and directions for future work need to be taken into account. Firstly, the small sample size fits the explorative purpose of our study. However, follow-up studies with larger groups are required to improve sensitivity to between-group differences, obtain insight into relations with clinical ratings of motor/cognitive impairments and evaluate test-retest reliability. Secondly, our findings are based on relatively mildly affected patients (see Table 1; mild cognitive and motor impairments). Participants had to be able to lift their arm above shoulder level, a requirement that cannot be fulfilled by a significant proportion of (subacute or chronic) stroke patients due to proximal paresis and/or synergies. Major changes to the current system are needed to also accommodate patients with more severely impaired upper extremity function. For example, arm support has to be allowed (e.g., lying on a table top) ─which requires a more extensive configuration of markers for environment tracking and may impact the accuracy of depth-image-based hand tracking─ and criteria for interaction with virtual objects have to be made adjustable to the patient’s capabilities (e.g., ‘touching’ or ‘grasping’) to avoid floor effects (and frustration) in severely affected patients and ceiling effects (and boredom) in mildly affected patients. Even then, the AR games will set some minimum requirements to the patient’s interaction space. Thirdly, this study was conducted with elderly participants. The current results therefore cannot be unreservedly generalized to younger persons, who are expected to deal with new technology with greater ease.

The patient-tailored and more natural interaction as well as the larger interaction space contributed to a good system usability that was considerably higher than in our previous study [15]. Inspection of SUS questions revealed that participants disagreed with Q2 (unnecessarily complex), Q6 (too much inconsistency) and Q8 (cumbersome to use). Only 10% of participants indicated that they needed to learn a lot before they could get going with the system (Q10) and 57% thought that most people would learn to use the system very quickly (Q7). Importantly, only 17% of participants indicated that they would *not* like to use the system frequently (Q1; 57% neutral, 27% positive). Inspection of presence questions taught us that participants were not distracted or hindered by technical issues, although ease and realism of object manipulation could be improved.

In conclusion, our study testifies to the potential of patient-tailored AR games for assessing motor impairments in patients with neurological conditions and provides starting points for further improvement. We envision that rapid technical developments will lead to higher accuracy of contactless hand tracking and to improvements in price, aesthetics (size, weight) and functionality (field of view, environment tracking without markers) of HMDs. However, many steps are still to be taken towards application in clinical practice (e.g. higher sensitivity to between-group differences, insight into relations with clinical ratings of motor/cognitive impairments, evaluation of test-retest reliability).

## Electronic supplementary material

ESM 1 **Online Resource 1** Presence questionnaire (PDF 79.1 kb)

ESM 2 **Online Resource 2** Technical details on experimental setup and AR games (PDF 262 kb)

ESM 3 **Online Resource 3** Illustrative video of game 1 “Balloons”. Two images are displayed on the laptop and in the screen captures: the left image is presented to the left eye and the right image is presented to the right eye to generate depth perception. The dark grey background appears almost transparent in the HMD so that the participant’s real hand and real environment (including the set of markers) are visible together with the virtual content (MP4 24.6 MB)

ESM 4 **Online Resource 4** Illustrative video of game 2 “Melody Cubes”. Two images are displayed on the laptop and in the screen captures: the left image is presented to the left eye and the right image is presented to the right eye to generate depth perception. The dark grey background appears almost transparent in the HMD so that the participant’s real hand and real environment (including the set of markers) are visible together with the virtual content (MP4 27.2 MB)

ESM 5 **Online Resource 5** Illustrative video of game 3 “Hungry Squirrel”. Two images are displayed on the laptop and in the screen captures: the left image is presented to the left eye and the right image is presented to the right eye to generate depth perception. The dark grey background appears almost transparent in the HMD so that the participant’s real hand and real environment (including the set of markers) are visible together with the virtual content (MP4 24.6 MB)

ESM 6 **Online Resource 6** Reachable workspace assessment (PDF 319 kb)

ESM 7 **Online Resource 7** Factors contributing to user experience (PDF 78.4 kb)

## References

[1] Hondori, H.M., Khademi, M., Dodakian, L., Cramer, S.C., and Lopes, C.V., A spatial augmented reality rehab system for post-stroke hand rehabilitation. In: Medicine Meets Virtual Reality, pp 279–285, 2013. 23400171

[2] Taske, A., Oppermann, L., Niemann, K., and Wilken, R., Design and Evaluation of a Stroke Rehabilitation Program. In: Virtuelle und Erweiterte Realität-12. Workshop der GI-Fachgruppe VR/AR. Shaker Verlag, pp 34–45, 2015.

[3] Burke JW, McNeill M, Charles DK, Morrow PJ, Crosbie JH, McDonough SM (2009). Optimising engagement for stroke rehabilitation using serious games. Vis. Comput..

[4] Luo, X., Kenyon, R.V., Kline, T., Waldinger, H.C., and Kamper, D.G., An augmented reality training environment for post-stroke finger extension rehabilitation. In: 9th International Conference on Rehabilitation Robotics (ICORR). IEEE, pp 329–332, 2005.

[5] Shen, Y., Ong, S., and Nee, A., Hand rehabilitation based on augmented reality. In: Proceedings of the 3rd International Convention on Rehabilitation Engineering & Assistive Technology. ACM, p 23, 2009.

[6] Alamri A, Cha J, El Saddik A (2010). AR-REHAB: An augmented reality framework for poststroke-patient rehabilitation. IEEE Trans. Instrum. Meas..

[7] Liu J, Mei J, Zhang X, Lu X, Huang J (2017). Augmented reality-based training system for hand rehabilitation. Multimedia Tools and Applications.

[8] Khademi, M., Hondori, H.M., Lopes, C.V., Dodakian, L., and Cramer, S.C., Haptic augmented reality to monitor human arm's stiffness in rehabilitation. In: 2012 IEEE EMBS Conference on Biomedical Engineering and Sciences (IECBES). IEEE, pp 892–895, 2012.

[9] Khademi, M., Mousavi Hondori, H., McKenzie, A., Dodakian, L., Lopes, C.V., and Cramer, S.C., Comparing direct and indirect interaction in stroke rehabilitation. In: CHI'14 Extended Abstracts on Human Factors in Computing Systems. ACM, pp 1639–1644, 2014.

[10] Mousavi Hondori H, Khademi M, Dodakian L, McKenzie A, Lopes CV, Cramer SC (2016). Choice of human–computer interaction mode in stroke rehabilitation. Neurorehabil. Neural Repair.

[11] Sousa, M., Vieira, J., Medeiros, D., Arsenio, A., and Jorge, J., SleeveAR: Augmented Reality for Rehabilitation using Realtime Feedback. In: Proceedings of the 21st International Conference on Intelligent User Interfaces. ACM, pp 175–185, 2016.

[12] Da Gama AEF, Chaves TM, Figueiredo LS, Baltar A, Meng M, Navab N, Teichrieb V, Fallavollita P (2016). MirrARbilitation: A clinically-related gesture recognition interactive tool for an AR rehabilitation system. Comput. Methods Prog. Biomed..

[13] Regenbrecht, H., McGregor, G., Ott, C., Hoermann, S., Schubert, T., Hale, L., Hoermann, J., Dixon, B., and Franz, E., Out of reach?—A novel AR interface approach for motor rehabilitation. In: 10th IEEE International Symposium on Mixed and Augmented Reality (ISMAR). IEEE, pp 219–228, 2011.

[14] Khademi, M., Mousavi Hondori, H., McKenzie, A., Dodakian, L., Lopes, C.V., and Cramer, S.C., Free-hand interaction with leap motion controller for stroke rehabilitation. In: CHI'14 Extended Abstracts on Human Factors in Computing Systems. ACM, pp 1663–1668, 2014.

[15] Cidota, M.A., Bank, P.J.M., Ouwehand, P.W., and Lukosch, S.G., Assessing Upper Extremity Motor Dysfunction Using an Augmented Reality Game. In: 2017 IEEE International Symposium on Mixed and Augmented Reality (ISMAR). IEEE, pp 144–154, 2017.

[16] Cidota, M.A., Lukosch, S.G., Bank, P.J.M., and Ouwehand, P.W., Towards Engaging Upper Extremity Motor Dysfunction Assessment Using Augmented Reality Games. In: 2017 IEEE International Symposium on Mixed and Augmented Reality (ISMAR-Adjunct). IEEE, pp 275–278, 2017.

[17] Hoehn MM, Yahr MD (1998). Parkinsonism: onset, progression, and mortality. Neurology.

[18] Marinus J, Visser M, Stiggelbout AM, Rabey JM, Martínez-Martín P, Bonuccelli U, Kraus PH, van Hilten JJ (2004). A short scale for the assessment of motor impairments and disabilities in Parkinson’s disease: the SPES/SCOPA. J. Neurol. Neurosurg. Psychiatry.

[19] van Swieten J C, Koudstaal P J, Visser M C, Schouten H J, van Gijn J (1988). Interobserver agreement for the assessment of handicap in stroke patients. Stroke.

[20] Fugl-Meyer AR, Jääskö L, Leyman I, Olsson S, Steglind S (1975). The post-stroke hemiplegic patient. 1. a method for evaluation of physical performance. Scand. J. Rehabil. Med..

[21] Hart, S.G., and Staveland, L.E., Development of NASA-TLX (Task Load Index): Results of empirical and theoretical research. In: Advances in psychology, vol 52. Elsevier, pp 139–183, 1988.

[22] IJsselsteijn, W., van den Hoogen, W., Klimmt, C., De Kort, Y., Lindley, C., Mathiak, K., Poels, K., Ravaja, N., Turpeinen, M., Vorderer, P., Measuring the experience of digital game enjoyment. In: Proceedings of Measuring Behavior, 2008. Noldus Information Tecnology Wageningen, Netherlands, pp 88–89, 2008.

[23] IJsselsteijn WA, de Kort YAW, Poels K (2013). The Game Experience Questionnaire.

[24] Brooke J (1996). SUS-A quick and dirty usability scale. Usability Evaluation in Industry.

[25] Gandy, M., Catrambone, R., MacIntyre, B., Alvarez, C., Eiriksdottir, E., Hilimire, M., Davidson, B., and McLaughlin, A.C., Experiences with an AR evaluation test bed: Presence, performance, and physiological measurement. In: 2010 9th IEEE International Symposium on Mixed and Augmented Reality (ISMAR). IEEE, pp 127–136, 2010.

[26] Cidota, M. A., Lukosch, S. G., Dezentje, P., Bank, P. J.M., Lukosch, H. K., and Clifford, R. M., Serious Gaming in Augmented Reality using HMDs for Assessment of Upper Extremity Motor Dysfunctions. I-Com 15(2):155–169, 2016.

[27] Field, A., Discovering statistics using IBM SPSS statistics. London, UK: SAGE Publications, 2013.

[28] Marsden C (1989). Slowness of movement in Parkinson's disease. Movement Disorders: Official Journal of the Movement Disorder Society.

[29] Bootsma RJ, Marteniuk RG, MacKenzie CL, Zaal FT (1994). The speed-accuracy trade-off in manual prehension: effects of movement amplitude, object size and object width on kinematic characteristics. Exp. Brain Res..

[30] Richard P, Birebent G, Coiffet P, Burdea G, Gomez D, Langrana N (1996). Effect of frame rate and force feedback on virtual object manipulation. Presence: Teleoperators & Virtual Environments.

[31] Levin MF, Magdalon EC, Michaelsen SM, Quevedo AA (2015). Quality of grasping and the role of haptics in a 3-D immersive virtual reality environment in individuals with stroke. IEEE Transactions on Neural Systems and Rehabilitation Engineering.

[32] Magdalon EC, Michaelsen SM, Quevedo AA, Levin MF (2011). Comparison of grasping movements made by healthy subjects in a 3-dimensional immersive virtual versus physical environment. Acta Psychol..

