# Supplementary material for: Patient-Tailored Augmented Reality Games for Assessing Upper Extremity Motor Impairments in Parkinson’s Disease and Stroke
Source: J Med Syst. 2018 Oct 30;42(12):246. doi: 10.1007/s10916-018-1100-9 (PMC6208648; doi:10.1007/s10916-018-1100-9)
Supplement: Supplementary file 1 — Online Resource 1 Presence questionnaire (PDF 79.1 kb) [file 10916_2018_1100_MOESM1_ESM.pdf]

Journal of Medical Systems

Patient-tailored augmented reality games for assessing upper extremity motor impairments in Parkinson's disease and stroke

Paulina J. M. Bank, PhD,* Marina A. Cidota, PhD, P. (Elma) W. Ouwehand, MSc., Stephan G. Lukosch, PhD

* Corresponding author: P.J.M. Bank, Department of Neurology, Leiden University Medical Center; PO Box 9600, 2300 RC Leiden, The Netherlands; E-mail: p.j.m.bank@lumc.nl Tel.: +31 71 526 3661; Fax: +31 71 524 8253. ORCID: 0000-0002-3127-398X

Online resource 1 - Questionnaire on presence (adapted from Gandy et al. 2010)

Presence total score is calculated as the summed score of all items (NB: items 1, 11, 15, 16, 20 and 21 have to be inverted).

Dutch version: *(NB: English translation below)*

**ERVARINGEN MET BEWEGEN IN DE VIRTUELE WERELD (PRESENCE)**	**Sterk mee oneens** ←			**Neutraal**		→	**Sterk mee eens**
1. Ik had het gevoel dat ik een toeschouwer was (ik had zelf weinig invloed op wat er gebeurde)	1	2	3	4	5	6	7
2. De bewegingen die ik heb gemaakt, lijken op de bewegingen die ik in het dagelijks leven maak om echte voorwerpen te verplaatsen	1	2	3	4	5	6	7
3. Ik was me erg bewust van gebeurtenissen in de virtuele omgeving	1	2	3	4	5	6	7
4. Ik kon goed voorspellen wat er zou gebeuren in reactie op mijn bewegingen	1	2	3	4	5	6	7
5. Ik kon de omgeving, voorwerpen en gebeurtenissen goed zien	1	2	3	4	5	6	7
6. Wanneer ik een *echt* voorwerp vastpakte of verplaatste, klopte dat wat ik voelde met hetgeen ik zag *(NB: alléén bij gebruik van echt voorwerp; niet van toepassing!)*	1	2	3	4	5	6	7

	Sterk mee oneens ⟵			Neutraal		⟶	Sterk mee eens
7. Ik kon goed inschatten waar de virtuele voorwerpen precies stonden	1	2	3	4	5	6	7
8. Ik kon de virtuele voorwerpen gemakkelijk vastpakken	1	2	3	4	5	6	7
9. Ik kon de virtuele voorwerpen goed verplaatsen	1	2	3	4	5	6	7
10. De bewegingen van het virtuele voorwerp klopten met de bewegingen van mijn hand	1	2	3	4	5	6	7
11. Er was veel vertraging tussen mijn eigen bewegingen en het verwachte resultaat	1	2	3	4	5	6	7
12. De bewegingen voelden natuurlijk aan	1	2	3	4	5	6	7
13. Aan het einde van het onderzoek, had ik het gevoel dat ik de taak onder de knie had.	1	2	3	4	5	6	7
14. Ik ging helemaal op in de taak	1	2	3	4	5	6	7
15. Bij het uitvoeren van de taak werd ik afgeleid of gehinderd door de *kwaliteit* van de beelden in de bril	1	2	3	4	5	6	7
16. De apparaten en technische spullen hebben mij gehinderd bij het uitvoeren van de taak.	1	2	3	4	5	6	7
17. Ik kon mij goed concentreren op het uitvoeren van de taak	1	2	3	4	5	6	7
18. Wat ik zag, klopte met wat ik hoorde en voelde.	1	2	3	4	5	6	7
19. De geluiden klopten met wat ik zag en voelde.	1	2	3	4	5	6	7
20. Er waren voorwerpen te zien die niet bij de taak pasten.	1	2	3	4	5	6	7
21. Tijdens de oefening zijn afbeeldingen of geluiden door de computer gemaakt, die niet realistisch waren.	1	2	3	4	5	6	7

English translation:

**PRESENCE**	**Strongly disagree** ⟵			**Neutral**		⟶	**Strongly agree**
1. I felt like an observer (I had little influence on what was happening)	1	2	3	4	5	6	7
2. Movements in the virtual environment were similar to daily-life reach-and-grasp movements	1	2	3	4	5	6	7
3. I was well aware of events occurring in the environment around me	1	2	3	4	5	6	7
4. I was well able to anticipate what would happen in response to my actions	1	2	3	4	5	6	7
5. I was well able to examine the environment, virtual objects, and events.	1	2	3	4	5	6	7
6. When I grasped or moved a real object, visual information was consistent with haptic information *(NB: only when a real object is used; not applicable in the current study)*	~~1~~	~~2~~	~~3~~	~~4~~	~~5~~	~~6~~	~~7~~
7. I was well able to estimate the position of virtual objects	1	2	3	4	5	6	7
8. I was well able to grasp the virtual objects	1	2	3	4	5	6	7
9. I was well able to move the virtual objects	1	2	3	4	5	6	7
10. Movements of the virtual object were congruent with movements of my hand	1	2	3	4	5	6	7
11. I experienced much delay between my actions and the expected outcomes	1	2	3	4	5	6	7
12. Movements felt natural	1	2	3	4	5	6	7
13. By the end of the experiment, I felt comfortable moving and interacting in the virtual environment	1	2	3	4	5	6	7

	Strongly disagree ⟵			Neutral			⟶ Strongly agree
14. I was "drawn in" to the experience	1	2	3	4	5	6	7
15. The visual display quality interfered or distracted me from performing the assigned tasks	1	2	3	4	5	6	7
16. The control devices interfered with the performance of the assigned tasks	1	2	3	4	5	6	7
17. I was well able to concentrate on the assigned tasks rather than on the mechanisms used to perform those tasks	1	2	3	4	5	6	7
18. The information provided through sight was consistent with my other senses	1	2	3	4	5	6	7
19. The information provided through sound was consistent with my other senses	1	2	3	4	5	6	7
20. There were objects in the virtual environment that didn't seem to belong there	1	2	3	4	5	6	7
21. During the experiment, the computer generated images or sounds were unrealistic	1	2	3	4	5	6	7
