# Supplementary material for: Patient-Tailored Augmented Reality Games for Assessing Upper Extremity Motor Impairments in Parkinson’s Disease and Stroke
Source: J Med Syst. 2018 Oct 30;42(12):246. doi: 10.1007/s10916-018-1100-9 (PMC6208648; doi:10.1007/s10916-018-1100-9)
Supplement: Supplementary file 2 — Online Resource 2 Technical details on experimental setup and AR games (PDF 262 kb) [file 10916_2018_1100_MOESM2_ESM.pdf]

Journal of Medical Systems

Patient-tailored augmented reality games for assessing upper extremity motor impairments in Parkinson's disease and stroke

Paulina J. M. Bank, PhD,* Marina A. Cidota, PhD, P. (Elma) W. Ouwehand, MSc., Stephan G. Lukosch, PhD

* Corresponding author: P.J.M. Bank, Department of Neurology, Leiden University Medical Center; PO Box 9600, 2300 RC Leiden, The Netherlands; E-mail: p.j.m.bank@lumc.nl Tel.: +31 71 526 3661; Fax: +31 71 524 8253. ORCID: 0000-0002-3127-398X

Online resource 2 – Technical details

Virtual content was visualized using an AIRO II head-mounted display (HMD; 2 OLED displays, each with a 1280x720 resolution, 16:9 aspect ratio and ≈ 40° diagonal FOV) (Cinoptics, Maastricht, The Netherlands), with Leap Motion for contactless tracking of the hand (Leap Motion Inc., San Francisco, CA, USA) and Logitech C922 Pro Stream Webcam for marker recognition mounted on top of it. The Leap Motion was used in its "head mounted display mode". The virtual world was aligned to the real world using the Vuforia tracking library (version 6.2.6, PTC Inc., Needham, USA), based on six A3 markers positioned in front of the patient at distances as shown in Fig. 1. A Microsoft Kinect^{TM} v2 sensor was placed at a height of 1.8 m and at a distance of approximately 3 meters from the participant, at an angle of 45° to the left side (to avoid occlusion by the markers).

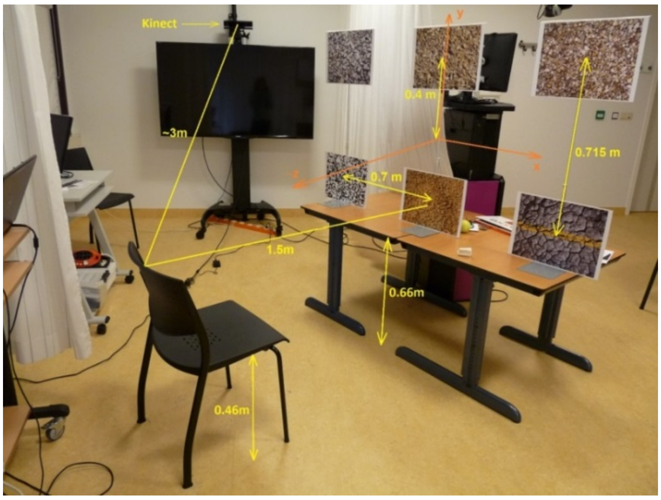

Fig. 1 Overview of the setup

In Fig. 1, the virtual coordinate system is represented by the orange axes, with the XOY plane being the plane of the markers. Distances were chosen so that during all experiments at least two markers at the same time were visible to the webcam mounted on the HMD. If one marker was occluded by the participant's hand, another marker was still recognized and provided the coordinate system for placing the virtual content. To ensure the best performance of Vuforia for marker detection and tracking, we considered the recommendations provided in [1]. For example, we used markers with a high-quality design (rated 5 out of 5 stars in the Vuforia Target Manager Database), we used A3 sized targets that were flat and not glossy-printed, and we performed the experiment under controlled lightning conditions (artificial, stable light). In our setup we obtained the best results by disabling the autofocus of the camera (in contrast to the suggestion in [1]), because the autofocus tended to keep focus on the hand rather than the markers, which negatively affected marker detection and tracking. In order to best translate the distances (units) in the real environment to our system, we chose a camera with low-distortion lens and the ratio of the printed targets was exactly the same with their digital correspondents.

Software components were integrated in Unity3D (version 5.6.0, Unity Technologies, San Francisco, USA). The Leap Motion Orion Beta software development kit (SDK) provided 3D-coordinates of hand 'joints' (e.g., hand palm and finger tips) at a sampling rate of 60 frames per second. The Kinect for Windows SDK (version 2.0) provided 3D-coordinates of body points (e.g., wrist, elbow and shoulder) at a sampling rate of 30 frames per second. The overall frame rate for the applications in Unity3D was variable (around 60 frames per second for games 1 and 2; 30 to 60 frames per second for game 3). Data from Kinect and Leap Motion were stored at this variable sampling rate, even though the Leap Motion sensor provided data at a higher sampling rate (up to ~110 frames per second). Prior to data processing, the recorded data were re-sampled to an evenly distributed time series (60 Hz).

The virtual scene was rendered from a first-person point of view camera, which was translated to a stereoscopic image for the left and the right eye. Using the parameters of the HMD (screen width, screen height) and "average measurements" for humans (e.g. approximately 6.4 cm distance between the user's eyes [2]), the position and the projection matrix of the stereo cameras were determined.

In all games, virtual objects had to be presented at a minimum distance of approximately 35 cm in front of the shoulder in order to keep a minimum distance from the Leap Motion that allowed for hand recognition (see game-specific descriptions). At this distance, no problems were encountered with the visualization of virtual content in the HMD. Virtual objects were mostly presented close to the 'maximum depth' of the

individually determined interaction space and all games involved movements away from the participant, towards a target close to or at maximum depth. Hence, games did not involve interaction with objects that were too close to the HMD for proper visualisation and/or interaction.

We built the virtual hand using the graphical hand model from our previous work [3], [4], available in the Unity package of Intel® Realsense SDK (version 7.0.23.8048), but with the joints' positions, orientations and rotations provided by the LeapMotion API. Only the ellipsoids representing the tips of the virtual index finger and thumb were visualized.

In all AR games, visual feedback was provided to facilitate interaction with the virtual content: a halo appeared when the virtual index finger touched the green line in game 1 (see video in Online Resource 3), virtual objects changed colour during interaction (see videos in Online Resources 4 and 5), and a visual cue was presented when the object of interest was located outside view of the HMD (i.e., a thick yellow line pointing from the HMD's center of view towards the virtual object; see videos in Online Resources 3 and 5).

Game 1: Balloons

At the beginning of game 1, the position of the head in the marker-based coordinate system was estimated. To this end, 5 positions of the AR virtual camera (i.e. the virtual correspondent of the webcam mounted on the top of HMD) were recorded during an interval of 5 seconds, while the patient was required to keep the head straight towards the markers, as still as possible (see video in Online Resource 3). The position of the head was the average of those five 3D coordinates. The position of the right/left shoulder was then determined relative to the position of the head as shoulder_position=head_position + (±0.17, -0.25, -0.19) (the unit in Unity represents one meter).

The four corners of the reachable interaction space were obtained by rotations around the estimated position of the shoulder of the tested arm. The rotation angles were mirrored for the left/right side (see Fig. 2). A balloon was placed at a faraway distance (approx. 2 m) on the 'forward' axis and was subsequently rotated around the 'right' axis (with -25° or 15°) and then around the 'up' axis (with -25° or 40°). The participants were asked to reach with the index finger as far as they could in the indicated direction and touch the green line between the balloon and their shoulder, while keeping the trunk against the backrest of the seat.

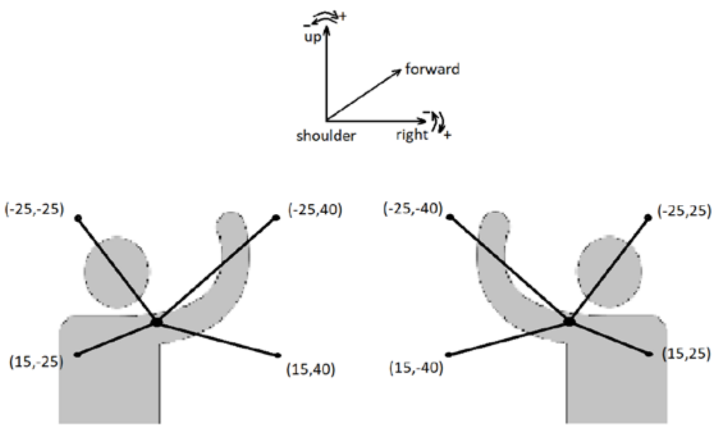

Fig. 2 Rotation angles (in degrees) around the estimated position of the shoulder, used for assessing the corners of the reachable interaction space

In the so obtained reachable interaction space, we defined a grid of 12 points (see Fig. 3) that was used to place the virtual content in all AR games. The depth of each point was computed by linear interpolation between the depths of the corners.

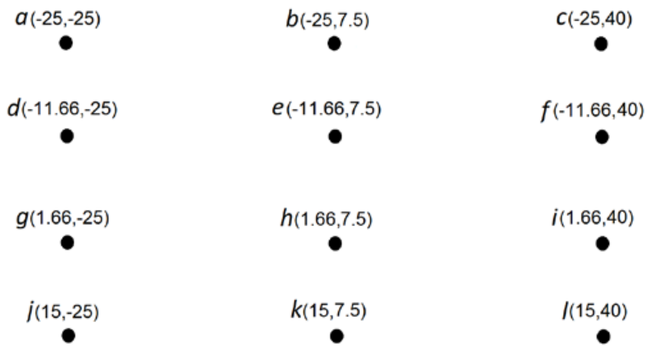

Fig. 3 Grid of 12 points (*a*-*l*) in the reachable interaction space, defined by rotation angles (in degrees) around the right shoulder. Depth of each point was computed by linear interpolation between the measured depths at the corners. Points from this grid were used to place virtual objects in *Game 1: Balloons* (part 2), in *Game 2: Melody Cubes* and in *Game 3: Hungry Squirrel*

In the second part of game 1, balloons were presented at random depths between 0.7 and 1.1 of the depth value of each point of the grid in Fig. 3, but not closer than approx. 35 cm from the shoulder[1]. Inside each balloon, a sphere with 4.5 cm in diameter was created to detect the touch of the index finger. Participants were instructed to touch the balloons as quickly as possible (see video in Online Resource 3). Balloons exploded upon touch, or disappeared if not touched within 20 seconds.

Game 2: Melody Cubes

Twelve opaque cubes (four of each size: 5, 7.5, and 10 cm) had to be moved from a stock pile located contralateral to the tested arm (from point *g* upwards to point *d* in Fig. 3; at 0.75 of the maximum depth, or at least approximately 35 cm from the shoulder)[1] to twelve empty cubes positioned at various positions within the interaction space central-ipsilateral to the tested arm (see video in Online Resource 4; the whole predefined ensemble of empty cubes had the bottom-center anchored at point *d* in Fig. 3, at maximum depth). Each opaque cube had an additional inner cube with a size of 0.5 of the (outer) cube size. Interaction was possible when the outer cube was touched with the thumb and the index finger, while neither of the fingers touched the inner cube. Ideally, the inner cube should be as large as possible to force participants to a specific hand opening size. However, in our implementation the inner cube size was rather small because otherwise the interaction would have been too difficult (i.e., too frequent loss of the cube during movement, perhaps also due to delay between real and virtual movement). When the cube fell within a size- and colour-matched empty cube (regardless of their respective orientations), cube placement was considered successful.

Game 3: Hungry Squirrel

Sixteen walnuts had to be put into a virtual basket that was alternately positioned in the upper or lower half of the interaction space (at points *b* or *k* in Fig. 3, respectively; at maximum depth), see video in Online Resource 5. At the beginning of each trial, one walnut appeared between the participant's shoulder and the basket (40 cm from the basket, or at least approximately 35 cm from the shoulder)[1]. The size of the walnut was approx. 6 cm,

[1] In all games, virtual objects had to be presented at a minimum distance of approximately 35 cm in front of the shoulder in order to keep a minimum distance from the Leap Motion that allowed for hand recognition. No adjustments were needed for game 1 or game 2. Only in game 3, the measured interaction space of twelve participants appeared insufficiently 'deep' to present the walnut 40 cm in front of the basket at the beginning of trials involving movements towards the upper and lower target (1 control, 5 PD patients and 2 stroke patients) or towards the upper target only (1 PD patients and 3 stroke patients). In these situations, the walnut appeared at the set minimum distance (approximately 35 cm) from the shoulder (i.e., at a slightly shorter distance from the basket).

the size of the basket was between 12.5-13 cm. To detect collision with the walnut, an invisible sphere (diameter approx. 5 cm) was placed around the head of the squirrel. In 8 out of 12 test trials, the obstacle was positioned halfway between the walnut's initial position and the basket. This squirrel was either visible from the start of the trial (visible obstacle, VO) or it appeared after the participant started moving the walnut (surprise obstacle, SO; 4x), i.e., as soon as the walnut was within a specific, individually adjusted distance from the squirrel's position (= estimated_speed * ART + 2.5 cm); estimated_speed for the upper/lower half was the average speed computed during the 3rd/4th 'practice' trial without obstacle; the available response time (ART) was set to 0.75 s. Unfortunately, this implementation of 'surprise obstacle' (SO) appeared not successful, due to between-trial variations of movement speed in combination with the relatively short movement distance appeared (limited by a minimum distance from HMD and the maximum reaching distance): if estimated_speed was high, the obstacle appeared almost immediately after the movement initiation, and SO looked almost like VO; if movement speed during a given trial was considerably higher than estimated_speed, there was not enough time for obstacle avoidance. Some participants therefore changed their movement strategy in anticipation and moved around the location of the possible obstacle, such that the 'trigger zone' for obstacle appearance was not touched, the obstacle did not appear, and SO looked like NO. Because some participants moved around the location of the obstacle, even if it was not supposed to appear at all (NO), our study underestimates the difference between NO and obstacle scenarios.

References

1. https://library.vuforia.com/articles/Solution/Optimizing-Target-Detection-and-Tracking-Stability.html

2. Dodgson NA (2004) Variation and extrema of human interpupillary distance. In: Stereoscopic Displays and Virtual Reality Systems XI, 2004. International Society for Optics and Photonics, pp 36-47

3. Cidota MA, Bank PJ, Ouwehand P, Lukosch SG (2017) Assessing Upper Extremity Motor Dysfunction Using an Augmented Reality Game. In: IEEE International Symposium on Mixed and Augmented Reality (ISMAR), 2017. IEEE, pp 144-154

4. Cidota MA, Clifford RM, Dezentje P, Lukosch SG, Bank PJ (2015) Affording Visual Feedback for Natural Hand Interaction in AR to Assess Upper Extremity Motor Dysfunction. In: IEEE International Symposium on Mixed and Augmented Reality (ISMAR), 2015. IEEE, pp 92-95
