# Supplementary material for: Patient-Tailored Augmented Reality Games for Assessing Upper Extremity Motor Impairments in Parkinson’s Disease and Stroke
Source: J Med Syst. 2018 Oct 30;42(12):246. doi: 10.1007/s10916-018-1100-9 (PMC6208648; doi:10.1007/s10916-018-1100-9)
Supplement: Supplementary file 6 — Online Resource 6 Reachable workspace assessment (PDF 319 kb) [file 10916_2018_1100_MOESM3_ESM.pdf]

Journal of Medical Systems

Patient-tailored augmented reality games for assessing upper extremity motor impairments in Parkinson's disease and stroke

Paulina J. M. Bank, PhD,* Marina A. Cidota, PhD, P. (Elma) W. Ouwehand, MSc., Stephan G. Lukosch, PhD

* Corresponding author: P.J.M. Bank, Department of Neurology, Leiden University Medical Center; PO Box 9600, 2300 RC Leiden, The Netherlands; E-mail: p.j.m.bank@lumc.nl Tel.: +31 71 526 3661; Fax: +31 71 524 8253. ORCID: 0000-0002-3127-398X

Online Resource 6: Reachable Workspace Assessment

Introduction

In AR game 1 (described in the main manuscript), each participant's interaction space was determined from the maximum reach distance (MRD) in four indicated movement directions, i.e., above/below the shoulder and at the ipsilateral/contralateral side of the body. Positions of virtual objects in the other AR games were based on this individually determined interaction space. Due to limited field of view of the head mounted device (HMD) in combination with the restricted volume for presentation of virtual content, however, it was not possible to use AR for measuring the true limits of one's reachable workspace in the vertical and horizontal direction. In this additional analysis, we therefore explore whether the MRD could serve as a proxy for the 3-dimensional upper extremity 'reachable workspace' in the frontal hemi-sphere, which may be considered a measure of overall functional status of the upper extremity. To this end, reachable workspace was evaluated in all participants prior to the AR games. Data collection and analysis were based on a procedure developed by Kurillo et al. [1], which has already been applied for evaluation of reachable workspace in several patient groups (e.g, pediatric and adult individuals with Duchenne or Becker muscular dystrophy [2] or fascioscapulohumeral muscular dystrophy [3], in individuals with musculoskeletal shoulder dysfunctions [4], and in individuals with amyotrophic lateral sclerosis [5]).

Methods

Measurement instruments

All measurement instruments are described in the main manuscript. Because the assessment of reachable workspace did not require interaction with the virtual environment, the Leap Motion was turned off and the participant was facing the Kinect™ v2 sensor (placed 1.8 m above the floor at 3 meters in front of the participant) instead of the markers (for spatial and temporal alignment of the virtual and real world, required for the AR games presented in the main manuscript). The AIRO II head mounted device (HMD) was used for presentation of instruction videos.

Data collection procedure

Participants sat in a chair without arm rests. Evaluation of reachable workspace was inspired by Kurillo et al. [1]. Participants performed a set of six movements, which consisted of lifting the arm from resting position (sitting upright with arms hanging down the sides) to above the head in vertical planes at around 0° (shoulder abduction), 45°, 90° (shoulder anteflexion), and 135°, and horizontal sweeps at the level of the umbilicus and shoulder. An instruction video was shown in the HMD prior to the start of each movement. Participants were instructed to reach as far as they could while keeping the elbow as extended as possible and the trunk against the backrest of the seat. If they were unable to reach further, they returned to the resting position and the next instruction video was started.

Data Analysis

Data was processed using MATLAB (The Mathworks Inc., Natick MA, USA, version R2016a).

Pre-processing

Data from Kinect was resampled to a 30Hz time series using linear interpolation before it was low-pass filtered (third-order Butterworth filter, cut-off frequency 5Hz) and transformed into a body-centric coordinate framework with origin in the shoulder joint (i.e., positions of wrist and elbow were expressed relative to the shoulder).

Step 1: Selecting data segments

Data segments containing the movements of interest were identified using a running window analysis on position of the wrist in *x*-, *y*-, and *z*-direction (Fig. 1).[1] The so obtained segments were visually inspected and their start and/or end time points were manually corrected if necessary. In addition, data points were eliminated if the arm was bent too much (i.e., distance between wrist and shoulder <70% of the average wrist-shoulder distance), which concerned on average 1.7% of data points per participant (median [IQR] = 0.9 [0-2.5] %; range: 0-8.6%).

Step 2: Determining the maximal boundaries (in spherical coordinate framework)

From here, we followed the procedure described by Kurillo et al. [1]. First, a spherical surface (with radius r_{mean}) was fitted into the remaining data points. Data were transformed from Cartesian coordinates (x, y, and z) into spherical coordinates (θ, φ and r). θ and φ corresponded to shoulder flexion/extension and abduction/adduction measurements, respectively [2], while r was set to r_{mean} for all data points (i.e., all data points were projected onto the fitted sphere). Maximal boundaries of the trajectory were determined by fitting a concave polygon to the data points using the alpha shape geometry with π/4 radius (Fig. 2). The boundary polygon was subsequently smoothed using Catmull-Rom splines.

[1] In specific, “resting episodes” between two consecutive movements were excluded from analysis by means of a running window analysis. Resting episodes were defined as data segments of at least 3 seconds in which the maximum position difference of the wrist was less than 10 cm in all three movement directions. Data points falling outside this range in any of the movement directions for >15 consecutive samples (i.e., 0.5 seconds) indicated movement onset and thus the end of a resting episode. The last 0.5 seconds prior to the detected movement onset and the first 0.5 seconds after movement offset (i.e., at the beginning of a resting episode) were included in the analysis. Because in some cases a resting episode of >3 seconds was detected *during* the movement of interest (i.e., at the maximum movement excursion), detected episodes were only excluded if the vertical position (z) was below the average z-position of the recording.

[2] Data points behind the shoulder (x<0) were projected to the frontal plane trough the shoulder (i.e., x=0) prior to transformation into spherical coordinates θ and φ.

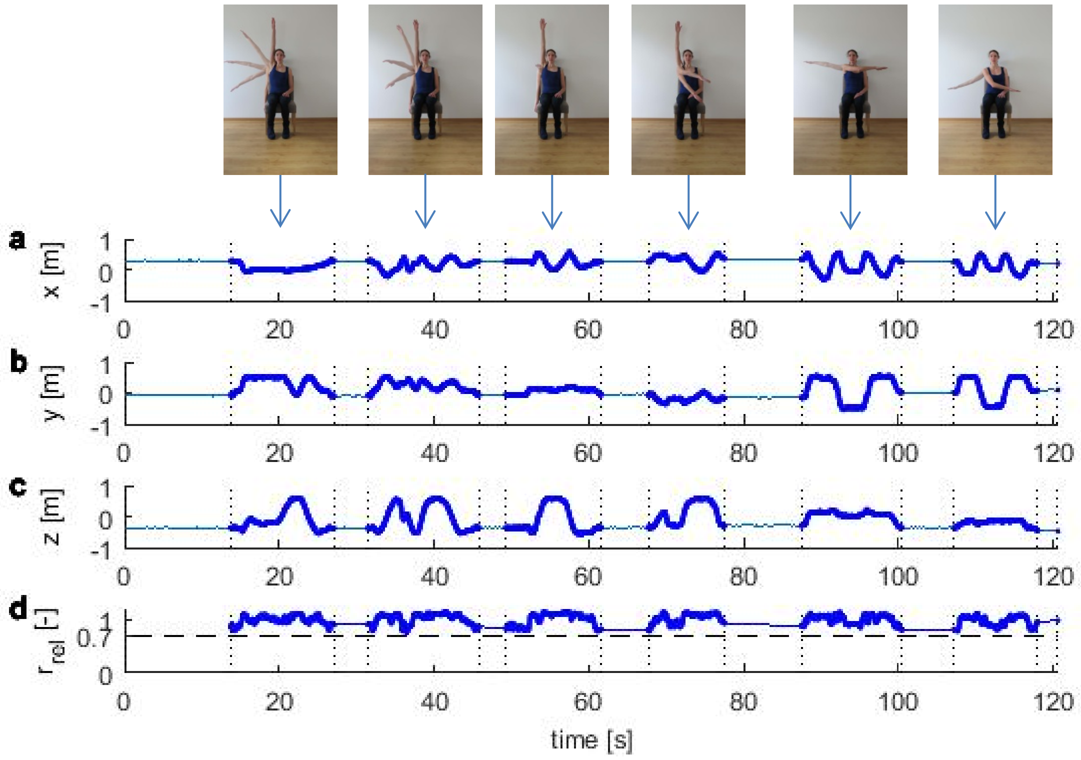

Fig. 1. *Step 1: Selection of data segments containing the movements of interest*; example based on data from a randomly selected participant (C019). A running window analysis on *x*, *y* and *z*-positions of the wrist relative to the shoulder (panels **a**, **b**, and **c**) was used to detect the beginning and end of resting episodes prior to, in between and after the six movements (see pictures above panel **a**). In all panels, dotted lines indicate the beginning and end of detected resting episodes. In addition, data points were eliminated if distance between wrist and shoulder <70% of the average wrist-shoulder distance (i.e., r_{rel} <0.7, panel **d**). Thick blue lines indicate the data points that were included in analysis based on the combination of these criteria.

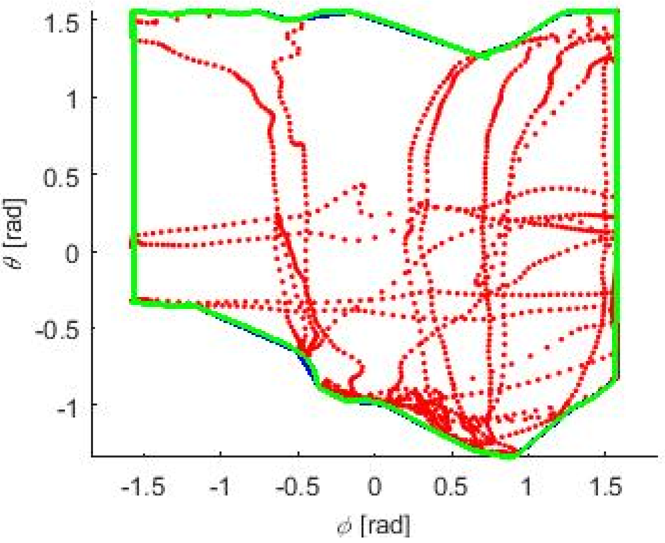

Fig. 2. *Step 2: Determining the maximal boundaries (in spherical coordinate framework)*; example based on data from participant C019. Selected data points were transformed from Cartesian coordinates (*x*, *y*, and *z*) into spherical coordinates (θ, φ and *r*, with *r* set to r_{mean} for all data points). The maximal boundaries enclosing these data points were determined by fitting a concave polygon (blue line) to the data points (red dots) using the alpha shape geometry with π/4 radius. The boundary polygon was subsequently smoothed using Catmull-Rom splines (green line).

Step 3: Calculating the relative workspace area

The smoothed boundary polygon was back-projected from spherical coordinates (θ, φ and r_{mean}) into Cartesian coordinates on the spherical surface (*x*, *y*, and *z*; Fig. 3). As described by Kurillo et al. [1], this spherical surface was divided in four quadrants that corresponded to clinically relevant functional subspaces (i.e., above/below the shoulder and at the ipsilateral/contralateral side of the body). For each quadrant, absolute workspace area (in m^2) was calculated by counting the number of patches on the spherical surface that fell inside the boundary polygon by the total number of patches on the spherical surface (i.e., 6144) and multiplying it by the total surface of the sphere (corresponding to $4\pi r_{mean}^2$). To allow comparison between individuals, the absolute workspace area was normalized by each individual's Kinect-extracted arm length (i.e., length of upper arm + forearm) and $1/4^{th}$ of a unit hemi-sphere. The so obtained *relative workspace area (RWA)* lies between 0 and 100% of the entire quadrant area.

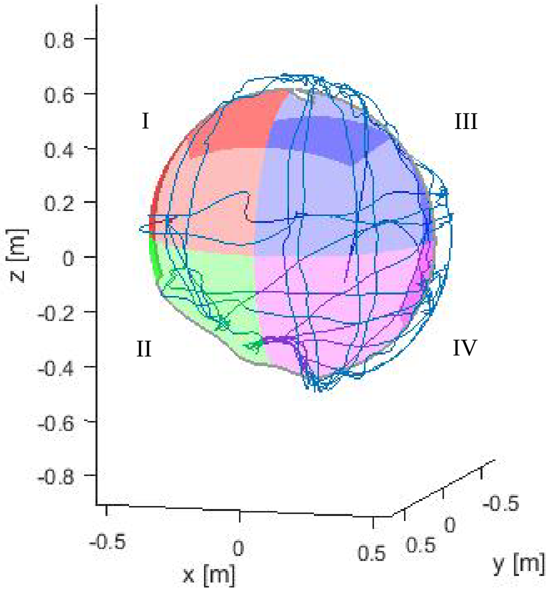

Fig. 3. *Step 3: Calculating the relative workspace area;* example based on data from participant C019 (tested arm: right) and inspired by graphical representation of Kurrilo et al. [1]. The original movement trajectories of the wrist (blue lines) are presented together with the spherical surface (with origin in the shoulder joint and radius r_{mean}) that was fitted to the selected data points (as determined in Step 1), and the smoothed boundary polygon enclosing all selected data points (as determined in Step 2, back-transformed to *x*, *y*, *z*-coordinates; grey line enclosing coloured surface). The relative workspace area (RWA, 0-100%) was calculated for each quadrant as the proportion of the surface within that quadrant that was enclosed by the boundary polygon. Red (I): above shoulder, contralateral side of the body; Green (II): below shoulder, contralateral side of the body; Blue (III): above shoulder, ipsilateral side of the body; Purple (IV): below shoulder, contralateral side of the body.

Statistical analysis

Two patients were excluded from statistical analysis of RWA (one stroke patient) and/or analysis of MRD (one stroke patient and one PD patient) due to technical issues with Kinect. Statistical analyses were performed using IBM® SPSS® Statistics 23.0 (IBM Corp., Armonk NY). Normality curves were inspected. RWA was submitted to mixed analyses of variance (ANOVAs) with group (PD vs. control, or stroke vs. control) as between-subjects factor and quadrant (ipsilateral upper, ipsilataral lower, contralateral upper, contralateral lower) as within-subjects factor. Degrees of freedom were adjusted if the sphericity assumption was violated [6]. Significance was set at $P<.05$, with Bonferroni correction for follow-up analyses. Effect size of significant main or interaction effects was quantified as partial eta squared (η_p^2). The potential association between RWA and MRD was

examined for each quadrant using Spearman's correlation coefficient (based on data from all 30 participants; non-parametric because substantial deviations from normality were observed for MRD).

Results

RWA tended to be slightly smaller in PD patients compared controls (53.6±13.7% vs. 64.3±8.2%; main effect of group: $F(1,18)=4.4$, $P=.05$, $\eta_p^2=.20$) and in stroke patients compared to controls (56.0±11.5% vs. 64.3±8.2%; $F(1,18)=3.5$, $P=.08$, $\eta_p^2=.16$). No significant interaction effects of group were observed. Post-hoc analysis of the main effect of quadrant (PD/control: $F(3,54)=71.3$, $P<.001$, $\eta_p^2=.74$; stroke/control: $F(1.7,30.4)=46.3$, $P<.001$, $\eta_p^2=.72$) revealed that RWA was smaller for contralateral quadrants (mean±SD over all participants, upper: 40.4±23.2%, lower: 37.1±10.7%) compared to ipsilateral quadrants (upper: 77.8±20.1%, lower: 76.6±10.3%), while there was no significant difference between quadrants above and below shoulder level on either side.

Significant correlations between RWA and MRD were observed for the ipsilateral quadrants (upper: $\rho=.55$, $P=.002$, lower: $\rho=.42$, $P=.03$), but not for the contralateral quadrants (upper: $\rho=.11$, $P=.57$; lower: $\rho=.25$, $P=.19$).

Discussion

Despite significant correlations between RWA and MRD for the ipsilateral quadrants, a comparison of our findings for RWA and MRD (as measured in AR game 1) suggests that MRD cannot be considered a proxy for the 3-dimensional upper extremity reachable workspace. While considerable between-participants variability was observed for RWA, there appeared to be ceiling effect for MRD in our sample of healthy controls and relatively mildly affected PD patients and stroke patients (who according to the inclusion criteria had to be able to lift their arms above shoulder level). It should be noted that the limited field of view of the HMD and space restrictions for aligning virtual content to the real world (despite the use of multiple markers) hampered evaluation of MRD in 'extreme' directions (see Online Resource 2 for technical details of AR game 1). As a consequence, values of MRD were close to 100% in all four indicated directions in all participants, with only very small differences between quadrants (ipsilateral upper quadrant: 98.0 ± 1.9%, ipsilateral lower quadrant 96.6±3.2%, contralateral lower quadrant: 97.0±2.5%, contralateral upper quadrant: 95.4±10.3%) and between groups (controls: 98.0±2.9%, PD patients: 96.8±2.9%, stroke patients: 95.5±2.9%). Although it seems plausible that reductions of MRD may be observed in severely affected patients with a considerably reduced upper extremity reachable workspace, the current study does not allow conclusions in this regard.

References

1. Kurillo G, Chen A, Bajcsy R, Han JJ (2013) Evaluation of upper extremity reachable workspace using Kinect camera. Technology and Health Care 21 (6):641-656

2. Han JJ, Kurillo G, Abresch RT, De Bie E, Nicorici A, Bajcsy R (2015) Upper extremity 3-dimensional reachable workspace analysis in dystrophinopathy using Kinect. Muscle & nerve 52 (3):344-355

3. Han JJ, Kurillo G, Abresch RT, Bie E, Nicorici A, Bajcsy R (2015) Reachable workspace in facioscapulohumeral muscular dystrophy (FSHD) by Kinect. Muscle & nerve 51 (2):168-175

4. Reddy D, Humbert S, Yu K, Aguilar C, de Bie E, Nicorici A, Kurillo G, Han J (2015) Novel Kinect-based method to assess 3D reachable workspace in musculoskeletal shoulder dysfunctions: AASE reports. Int J Phys Med Rehabil 3 (274):2

5. Oskarsson B, Joyce NC, De Bie E, Nicorici A, Bajcsy R, Kurillo G, Han JJ (2016) Upper extremity 3-dimensional reachable workspace assessment in amyotrophic lateral sclerosis by Kinect sensor. Muscle & nerve 53 (2):234-241

6. Field A (2013) Discovering statistics using IBM SPSS statistics. SAGE Publications.
