# Supplementary material for: Patient-Tailored Augmented Reality Games for Assessing Upper Extremity Motor Impairments in Parkinson’s Disease and Stroke
Source: J Med Syst. 2018 Oct 30;42(12):246. doi: 10.1007/s10916-018-1100-9 (PMC6208648; doi:10.1007/s10916-018-1100-9)
Supplement: Supplementary file 7 — Online Resource 7 Factors contributing to user experience (PDF 78.4 kb) [file 10916_2018_1100_MOESM4_ESM.pdf]

Journal of Medical Systems

Patient-tailored augmented reality games for assessing upper extremity motor impairments in Parkinson's disease and stroke

Paulina J. M. Bank, PhD,* Marina A. Cidota, PhD, P. (Elma) W. Ouwehand, MSc., Stephan G. Lukosch, PhD

* Corresponding author: P.J.M. Bank, Department of Neurology, Leiden University Medical Center; PO Box 9600, 2300 RC Leiden, The Netherlands; E-mail: p.j.m.bank@lumc.nl Tel.: +31 71 526 3661; Fax: +31 71 524 8253. ORCID: 0000-0002-3127-398X

Online Resource 7: Factors contributing to user experience

As described in the main paper, we aimed to further our understanding of factors contributing to user experience. To this end, we explored the associations between the different aspects of user experience (i.e., task load, engagement, usability and presence), and associations between aspects of user experience and 'general' measures of performance on the AR games. Although it would also be interesting to examine potential relations between clinical characteristics (i.e., disease severity, motor/cognitive impairments) and user experience or AR outcomes, the sample size of this study is too small for a meaningful evaluation of correlations within separate patient groups.

Methods

As described in the main paper, the perceived task load was evaluated upon completion of each game using the NASA-TLX questionnaire (1-7; high: worse [1]) and engagement was evaluated using a subset of 14 questions (1-5; high: better [2]) from the Game Experience Questionnaire (GEQ; [3,4]). At the end of the experiment, the System Usability Scale (SUS; 0-100; high: better [5]) and a questionnaire on presence (1-7; high: better; Online Resource 1; adapted from [6]) were used to evaluate user experiences and identify opportunities for further improvement.

Because the analyses presented in the main paper revealed no group differences for any of these questionnaires, data from the three participant groups were combined in order to explore whether user experiences (i.e., task load, engagement, usability and presence) were associated with 'general' game characteristics (i.e., success rates, time per object). All associations were explored using Spearman's correlation

coefficient ρ. Significance was set to $P<.05$. Because this was regarded as an exploratory analysis, statistical significance was not adjusted for multiple testing [7].

Results

It can be appreciated from Table 1 that higher scores for usability and presence tended to be associated with lower scores for task load and higher scores for engagement. In addition, a significant correlation was observed between usability and presence ($\rho=.62$, $P<.001$). Table 2 reveals that longer time per object appeared to be associated with higher task load (significant for games 2 and 3) and lower presence (significant for game 3).

Table 1. Correlations between user experiences

	Task load (NASA-TLX)	Engagement (GEQ-subset)	Usability (SUS)	Presence
Game 1: Balloons				
Task load (NASA-TLX)	.	-.01	-.29	-.27
Engagement (GEQ-subset)	-.01	.	.33	.46*
Game 2: Melody Cubes				
Task load (NASA-TLX)	.	-.18	-.36	-.47**
Engagement (GEQ-subset)	-.18	.	.28	.46*
Game 3: Hungry Squirrel				
Task load (NASA-TLX)	.	-.22	-.44*	-.52**
Engagement (GEQ-subset)	-.22	.	.18	.47**

Based on $N=30$ participants (10 controls, 10 stroke patients and 10 PD patients). * $P<.05$, ** $P<.01$.

Table 2. Correlations between user experiences and general AR game characteristics

	Task load (NASA-TLX)	Engagement (GEQ-subset)	Usability (SUS)	Presence
Game 1: Balloons				
Success rate	-.02	-.35	-.09	-.11
$T_{balloon}$	.27	-.14	-.26	-.21
Game 2: Melody Cubes				
T_{cube}	.37*	.09	.07	-.18
Game 3: Hungry Squirrel				
Success rate (scenario VO)	-.26	.01	.10	.01
T_{walnut}	.52**	-.23	-.22	-.41*

Based on N=30 participants (10 controls, 10 stroke patients and 10 PD patients). * P<.05, ** P<.01.

References

1. Hart SG, Staveland LE (1988) Development of NASA-TLX (Task Load Index): Results of empirical and theoretical research. In: Advances in psychology, vol 52. Elsevier, pp 139-183

2. Cidota MA, Bank PJ, Ouwehand P, Lukosch SG (2017) Assessing Upper Extremity Motor Dysfunction Using an Augmented Reality Game. In: IEEE International Symposium on Mixed and Augmented Reality (ISMAR), 2017. IEEE, pp 144-154

3. IJsselsteijn W, Van Den Hoogen W, Klimmt C, De Kort Y, Lindley C, Mathiak K, Poels K, Ravaja N, Turpeinen M, Vorderer P (2008) Measuring the experience of digital game enjoyment. In: Proceedings of Measuring Behavior, 2008. Noldus Information Technology Wageningen, Netherlands, pp 88-89

4. IJsselsteijn WA, de Kort YAW, Poels K (2013) The Game Experience Questionnaire. Eindhoven: Technische Universiteit Eindhoven.

5. Brooke J (1996) SUS-A quick and dirty usability scale. Usability evaluation in industry 189 (194):4-7

6. Gandy M, Catrambone R, MacIntyre B, Alvarez C, Eiriksdottir E, Hilimire M, Davidson B, McLaughlin AC (2010) Experiences with an AR evaluation test bed: Presence, performance, and physiological measurement. In: 9th IEEE International Symposium on Mixed and Augmented Reality (ISMAR), 2010. IEEE, pp 127-136

7. Bender R, Lange S (2001) Adjusting for multiple testing—when and how? Journal of clinical epidemiology 54 (4):343-349
